# New Books

**Published:** 2010-02

**Adaptation to Climate Change: A Spatial Challenge**

Rob Roggema

New York:Springer, 2010. 250 pp.

ISBN: 978-1-4020-9358-6, $129

**An Introduction to Mathematical Models in Ecology and Evolution: Time and Space, 2nd ed.**

Mike Gillman

Hoboken, NJ:John Wiley & Sons, 2009. 168 pp. ISBN: 978-1-4051-9489-1, $130

**Bioinformatics: Tools and Applications**

David Edwards, Jason Stajich, David Hansen, eds.

New York:Springer, 2009. 451 pp.

ISBN: 978-0-387-92737-4, $89.95

**Climate Change Adaptation in New York City: Building a Risk Management Response**

New York City Panel on Climate Change, ed.

Hoboken, NJ:John Wiley & Sons, 2010. 328 pp. ISBN: 978-1-57331-800-6, $130

**Ecological Economics Reviews**

Karin E. Limburg, Robert Costanza, Ida Kubiszewski, ed.

Hoboken, NJ:John Wiley & Sons, 2010. 352 pp. ISBN: 978-1-57331-766-5, $130

**Energy Demand and Climate Change: Issues and Resolutions**

Franklin Hadley Cocks

Hoboken, NJ:John Wiley & Sons, 2009. 267 pp. ISBN: 978-3-527-32446-0, $37.50

**Environmental Ethics: The Big Questions**

David R. Keller, ed.

Hoboken, NJ:John Wiley & Sons, 2010. 608 pp. ISBN: 978-1-4051-7639-2, $99.95

**Evaluation of Certain Food Additives**

World Health Organization

Geneva:WHO Press, 2009. 219 pp.

ISBN: 978-9-2412-0952-6, $40

**Fundamentals in Air Pollution: From Processes to Modelling**

Bruno Sportisse

New York:Springer, 2010. 299 pp.

ISBN: 978-90-481-2969-0, $89.95

**Global Governance of Hazardous Chemicals: Challenges of Multilevel Management**

Henrik Selin

Cambridge, MA:MIT Press, 2010. 240 pp. ISBN: 978-0-262-01395-6, $44

**Mining, Society, and a Sustainable World**

Jeremy Richards, ed.

New York:Springer, 2010. 506 pp.

ISBN: 978-3-642-01102-3, $169

**Pricing Nature: Cost–Benefit Analysis and Environmental Policy**

Nick Hanley, Edward B. Barbier

Northhampon, MA:Edward Elgar, 2009. 360 pp. ISBN: 978-1-84542-789-4, $144

**Safety Evaluation of Certain Food Additives**

World Health Organization

Geneva:WHO Press, 2009. 639 pp.

ISBN: 978-9-2416-6060-0, $150

**Science and Sustainable Food Security**

M.S. Swaminathan

Hackensack, NJ:World Scientific, 2009. 436 pp. ISBN: 978-981-4282-10-9, $98

**The Wetlands Handbook**

Edward Maltby, Tom Barker, eds.

Hoboken, NJ:John Wiley & Sons, 2009. 800 pp. ISBN: 978-0-632-05255-4, $300

**Tools, Techniques and Approaches for Sustainability**

William R Sheate

Hackensack, NJ:World Scientific, 2009. 420 pp. ISBN: 978-981-4289-68-9, $82

**Toxicological Evaluation of Certain Veterinary Drug Residues in Food**

World Health Organization

Geneva:WHO Press, 2009. 240 pp.

ISBN: 978-9-2416-6061-7, $70

**Uncertainty Modeling in Dose Response: Bench Testing Environmental Toxicity**

Roger M. Cooke

Hoboken, NJ:John Wiley & Sons, 2009. 230 pp. ISBN: 978-0-470-44750-5, $110

**Water**

John Knechtel, ed.

Cambridge, MA:MIT Press, 2009. 320 pp. ISBN: 978-0-262-01329-1, $15.95

